# Node Scheduling Strategies for Achieving Full-View Area Coverage in Camera Sensor Networks

**DOI:** 10.3390/s17061303

**Published:** 2017-06-06

**Authors:** Peng-Fei Wu, Fu Xiao, Chao Sha, Hai-Ping Huang, Ru-Chuan Wang, Nai-Xue Xiong

**Affiliations:** 1 School of Computer Science, Nanjing University of Posts and Telecommunications, Nanjing 210003, China; 2014070240@njupt.edu.cn (P.-F.W.); xiaof@njupt.edu.cn (F.X.); hhp@njupt.edu.cn (H.-P.H.); wangrc@njupt.edu.cn (R.-C.W.); 2 Jiangsu High Technology Research Key Laboratory for Wireless Sensor Networks, Nanjing 210003, China; 3 Nanjing University of Information Science & Technology, Nanjing 210044, China; 4 Department of Mathematics and Computer Science, Northeastern State University, Tahlequah, OK 74464, USA; xiongnaixue@gmail.com

**Keywords:** full-view area coverage, camera sensor networks, sleep scheduling

## Abstract

Unlike conventional scalar sensors, camera sensors at different positions can capture a variety of views of an object. Based on this intrinsic property, a novel model called full-view coverage was proposed. We study the problem that how to select the minimum number of sensors to guarantee the full-view coverage for the given region of interest (ROI). To tackle this issue, we derive the constraint condition of the sensor positions for full-view neighborhood coverage with the minimum number of nodes around the point. Next, we prove that the full-view area coverage can be approximately guaranteed, as long as the regular hexagons decided by the virtual grid are seamlessly stitched. Then we present two solutions for camera sensor networks in two different deployment strategies. By computing the theoretically optimal length of the virtual grids, we put forward the deployment pattern algorithm (DPA) in the deterministic implementation. To reduce the redundancy in random deployment, we come up with a local neighboring-optimal selection algorithm (LNSA) for achieving the full-view coverage. Finally, extensive simulation results show the feasibility of our proposed solutions.

## 1. Introduction

Research on wireless sensor networks has aroused explosive interest since it can be the bridge between the physical world and digital information [1]. With the improvements in microelectro-mechanical systems (MEMS) technology, multimedia capability has been added to sensor nodes which can thus provide more detailed and exciting information about the environment [2,3,4]. As a result researchers have recently started to work on wireless multimedia sensor networks. 

Compared with scalar sensors the current multimedia sensors such as camera sensors and ultrasound sensors have an important characteristic, namely directionality. Wireless multimedia sensor networks are also variously known as directional sensor networks (DSNs), visual sensor networks (VSNs) or camera sensor networks (CSNs). Visual coverage is an important quantifiable property of camera sensor networks, describing the visual data collection within the sensing range of the systems. The knowledge of visual coverage guides the deployment of the whole network. Traditional signal-based sensor networks cannot identify differences between similar targets, while the visual information has a more inherent advantage in this respect [5]. Consequently, there are numerous applications employing camera sensor networks to monitor the environment, like maritime oil exploration, intruder recognition and tracking, military and civilian activity monitoring, etc.

It is critical to capture in real-time the easily identified posture of intruders for the sake of the recognition efficiency. For example, a camera can precisely identify a target only if he/she is in front of or near the camera; that is, the target is facing straight into the camera. However, in practical applications the face direction of intruders is uncertain, which represents a significant challenge for camera sensor networks. To solve this problem, the full-view coverage model has been proposed to guarantee the face recognition of intruders from any arbitrary direction. If no matter which direction the target is facing there is always a sensor whose sensing range includes the target and the sensor′s viewing direction is sufficiently close to the direction the target is facing, the target is considered as being full-view covered. Similar to the full-view coverage, the *k*-coverage model overlaps the sensing range of sensors with the purpose of improving the fault-tolerance of the network [6]. Compared with the *k*-coverage model, the full-view coverage model has more pertinence to the object’s facing direction.

Just like the coverage issue in wireless sensor networks, the geometric shape of the monitored target can be seen as a linear region, and then be developed in the two-dimensional plane. Full-view coverage problems can be classified as target-based coverage [7,8,9,10,11,12], barrier-based coverage [13,14], and area-based coverage [15,16], with the complexity of the research gradually increasing. In this paper, we focus on the area-based full-view coverage problem. Informally, if the camera sensor network monitors the ROI from all perspectives, it is guaranteed that every aspect of an object at any position can be captured by the camera nodes in the CSN.

One significant problem in full-view area coverage is how to decrease the complexity of the issue caused by the irregular sensing range and the variable viewing direction. It was proved that the full-view area coverage with a minimum number of sensors (FACM) problem is NP-hard [17,18]. Existing works commonly use approximation algorithms to solve it [15,19,20,21]. Another important question is how to deduce the critical sensor density needed both in deterministic and random deployment for full-view coverage. Some references [18,22,23,24] have given some results for the critical density, which believe that there exists asymptotic coverage for mathematical convenience when the total number of cameras approaches to infinity. Our main contributions are highlighted as follows.

- We deduce the necessary and sufficient conditions of the FACM problem for the local point and study the geometric parameters, the maximum full-view neighborhood coverage and the position trajectory of sensors around the local point.
- We find that the full-view area coverage can be guaranteed approximately, as long as the regular hexagons decided by the virtual grids are seamlessly stitched.
- We propose two solutions for camera sensors for two different deployment strategies, respectively. By computing the theoretical optimal length of the virtual grids, the deployment pattern algorithm (DPA) for FACM is presented in the deterministic implementation. For reducing the redundancy in random deployment, a local neighboring-optimal selection algorithm (LNSA) is devised for achieving the full-view coverage in the grid points.

The paper is organized as follows: we discuss the works of literature related to the full-view coverage in Section 2. Then the geometric analysis and preliminaries based on the network model are described in Section 3. We derive the density estimation and the deployment pattern algorithm (DPA) for deterministic implementation in Section 4. However, a local neighboring-optimal selecting algorithm (LNSA) is proposed for full-view area coverage in random implementation in Section 5. The feasibility of the proposed DPA and LNSA is demonstrated with simulation results in Section 6. We finally conclude the paper and discuss the future work in Section 7.

## 2. Related Work

For improving the quality of service (QoS) of CSNs, researchers have recently dedicated much effort to the coverage issue in sensor networks. Since extensive works based on omnidirectional sensing models have not been applied to CSNs, the analysis of coverage models in camera sensor networks has received sustained attention [25,26,27,28,29]. Mavrinac [30] analyzed available coverage models in the context of specific applications for CSNs and derived a generalized geometric model. In allusion to the intrinsic nature of camera sensors, the binary sector coverage model has been widely adopted in the existing works. Guvensan et al. [31] listed all properties of camera sensor nodes and concluded the five enhancement principles [5,15,32,33,34,35]. It is obvious to find that the existing research had focused on two general solutions: adjusting the working directions of the sensor nodes and scheduling the sleep durations of redundant sensors.

The previous works often focus on the coverage enhancement by scheduling the sensor nodes themselves. When researchers started to take the object’s face direction into consideration, the coverage issue in camera sensor networks became more complicated and exciting. Wang and Cao [36] introduced the concept of full-view coverage and derived the conditions for guaranteeing the full-view coverage of a point or a sub-region. Moreover, they obtained the sensor density needed for full-view coverage both in random deployment and triangular lattice-based implementation. Inspired by [36], we study the sufficient and necessary condition of the full-view point coverage under the constraint with a minimum number of camera sensors.

Hu et al. [18] defined a centralized parameter—equivalent sensing radius (ESR)—to evaluate the critical requirement for asymptotic full-view coverage under uniform deployment in heterogeneous CSNs. They investigated the impact of mobility and present four mobility models. Besides, they first took heterogeneity of camera sensors into consideration, since camera sensors may come from different manufacturers and have different sensing parameters, or experience different obstruction by the terrain. The ESR of four mobility models may be derived with the statistical method, which assumes that the number of camera nodes tends toward infinity. 

Similar to [18], Wu [37] also defined a centralized parameter—critical sensing area (CSA)—to evaluate the critical requirement for asymptotic full-view coverage in heterogeneous networks. They investigated the full-view coverage problem under uniform deployment and Poisson deployment. Here the statistical method was also accepted for computing the probabilities that all points in a dense grid meet the conditions of full-view coverage. The work of [18,37] is conducive to understanding the influence between full-view coverage quality and the parameters of camera nodes, such as sensing radius, number of sensors and the effective angle.

He and co-workers [18] studied how to select the minimum number of camera sensors to guarantee the full-view coverage of a given region. Inspired by the work of [36], they innovatively adopted dimension reduction method to decrease the complexity of the FACM problem. Here the minimum-number full-view point coverage is NP-hard. They solved the NP-hard problem by two approximation algorithms, i.e., the greedy algorithm (GA) and the set-cover-based algorithm (SCA).

The full-view coverage was extended from 2-D plane to the 3-D surface by Manoufali et al. [38], whose algorithm has high complexity for the reason that they take the effect of realistic sea surface movement into consideration. Besides, they proposed a method to improve the target detection and recognition in the presence of poor link quality using cooperative transmission with low power consumption. Similar work also was proceeded by Xiao [39], who studied the coverage problem in directional sensor networks for 3-D terrains and designed a surface coverage algorithm. The sensing direction of camera nodes was uniformly set as vertically downward, which helps give the profile map of the area covered by nodes in the ideal case. Although research on 3-D surface coverage in CSNs is a recent emerging topic, it is not the focus of this paper. 

As one branch of the full-view coverage, the face-view barrier coverage problem is also presented in the recent literature [13,40,41,42]. Ma [13] defined the minimum camera barrier coverage problem (MCBCP) for achieving full-view coverage in the barrier field. Their main contribution could be summarized as modeling the weighted directed graph and proposing an optimal algorithm for the MCBCP problem. Different from the barrier coverage described in [13], Yu [40] paid more attention to the intruderS′ trajectory along the barrier and head rotation angles. They derived a rigorous probability bound for intruder detection via a feasible deployment pattern, which could guarantee the intruderS′ face always been detected in the barrier.

Zhao [41] considered the power conservation and addressed the efficient sensor deployment (ESD) problem and energy-efficient barrier coverage (EEBC) problem. They solved this optimization problem and proposed a scheduling algorithm to prolong the network lifetime. Tao [42] summarized the sensing properties and behaviors of directional sensors in the majority of studies on barrier coverage and classified the existing research results. The barrier coverage issue is the special case of the area coverage which means the shape of the covered field is a fence. Since the main difference between barrier-based coverage and area-based coverage is the geometric shape of the monitored target, which is decided by the modeling of the specific applications, we focus on the issue of the full-view area coverage. In this paper, we deduce the necessary and sufficient conditions of the FACM problem from a novel perspective. DPA and LNSA are proposed for two implementation strategies. We compare our conclusions mainly with [19,36], whose results are mostly relevant to our works. We list the comparison between our work and the past related works in Table 1.

## 3. Problem Description and Assumptions

### 3.1. Network Model

As mentioned in the Introduction, the sector sensing model has been adopted in many studies [13,15,18,19,24,26,36,37,38]. In this paper, we also adopt the sector sensing model and explain this model in the following content. Given a set of camera nodes is deployed over a field without any obstacles, we assume that the camera nodes are homogeneous with the same sensing radius and the angle of view. The region sensed by a camera node can be presented as a 4-tuple $\langle S,\vec{d},R,2\varphi \rangle$ including the position *S*, the working direction $\vec{d}$, the sensing radius *R*, and the angle of view 2φ. We assume the intruder can be represented as a 2-tuple $\langle p,\vec{f}\rangle$ with the location *P* and the facial direction $\vec{f}$. 

**Figure 1:**
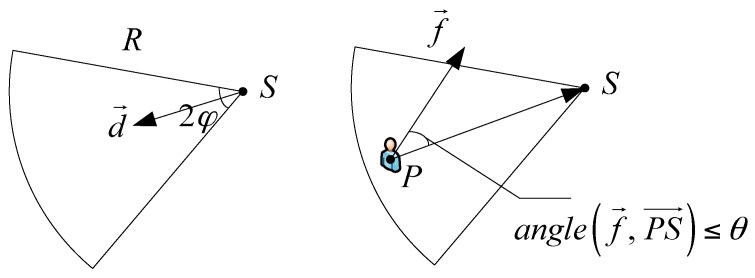
The sensing model of the camera node.

The sensing capability of detecting the intruder is illustrated in Figure 1. There exists a predefined parameter which is called the effective angle θ. If the angle between the object’s facing direction $\vec{f}$ and the vector $\vec{PS}$ exceeds the effective angle θ, we assume that the sensor cannot recognize the intruder, even if the location *P* is located in the field of view *Sc*. 

The relationship of the sensor position *S*, the sensor working direction $\vec{d}$, and the intruder location *P* can be determined by an Intruder in Sector (IIS) test. We state the intruder passes the IIS test if and only if it meets two conditions as follows:

- The one condition is that the intruder location *P* must fall within the field of view sensed by the camera node, which is donated by P∈Sc.
- The one other condition is that the angle between the object’s facing direction and the camera’s working direction is less than or equal to the effective angle, i.e., $angle\left(\vec{f},\vec{PS}\right)\leq \theta$.

To facilitate reading, we provide in Table 2 a glossary of the variables that will be utilized throughout this paper.

### 3.2. Definition and Problem Description

To capture the facial image of the intruder no matter which direction he is facing towards, the concept of full-view coverage has been proposed for CSNs. Based on the definitions of full-view coverage in previous works, we further give two principal definitions and the main full-view coverage problem which will be solved in the following sections.

**Definition 1** (View coverage and full-view point coverage). *For an intruder P with facial direction* $\vec{f}$ *appearing in ROI, there is always a camera node S making the intruder pass the IIS test. We state that the intruder is view covered by node S. The intruder is full-view covered if and only if there always is a node making the intruder pass IIS test for all possible face directions.*

View coverage is more generalized coverage model which has drawn attention in [21,40]. It is often used in the constrained condition such as in barrier coverage where the intruders pass through a bounded region from the entrance to the exit with all continuous paths. This is not the focus of our paper.

**Definition 2** (Full-view area coverage problem)**.** Without loss of generality, the intruder mentioned above can be seen as a point. Each point of the region is also important in the area-based coverage. A region is full-view covered if every point in it is full-view covered.

With the definition mentioned above, the FACM problem can be stated as follows:

*Given*: *n* homogenous camera sensors are deployed in a ROI *A*.

*Problem*: Find a set of camera sensors and make ROI be full-view covered by a minimum number of sensors. It can also be further elaborated the problem under two situations. Under the deterministic deployment strategy, it can be described as computing the critical density and positions of sensors for achieving full-view area coverage. Under the random deployment strategy, the work mainly focuses on adjusting sensorS′ working direction and scheduling the sleep durations of redundant sensors.

### 3.3. Preliminaries

**Figure 2:**
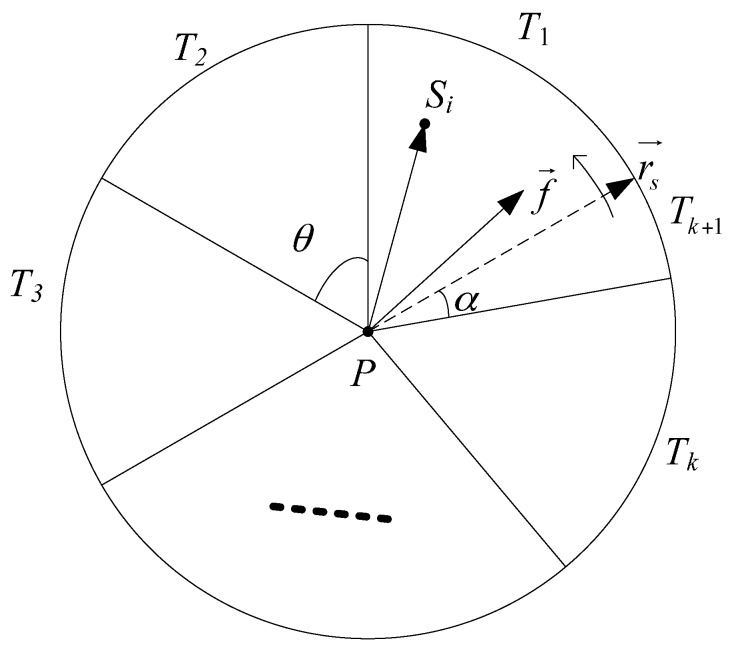
The sufficient condition for full view coverage of an arbitrary point *P.*

Firstly, we develop the sufficient condition for full view coverage of an arbitrary point *P* in the region *A*. As Figure 2 shows, *D*(*P*, *R*) is a disk centered at *P* with radius *R*. Without loss of generality, denote the dashed radius $\vec{r_{s}}$ as the start line. Rotate the start line anti-clockwise with θ to get sector *T*_1_, *T*_2_, …, *T_k_*, $k=\left\lfloor \frac{2\pi }{\theta }\right\rfloor$. If $k<\frac{2\pi }{\theta }$, then between *T*_1_ and *T_k_* there may be one more sector *T_k_*_+1_ with the interior angle α∈(0, θ). In the following explanation of the sufficient condition, we agree on $k<\frac{2\pi }{\theta }$.

The sufficient condition about a point *P* to be full-view covered is such that, at least one node *S_j_* locates in *T_j_* and makes *P* pass IIS test, *j* = 1, 2, ···, *k* + 1. The proof is simple [37]. No matter which the facial direction $\vec{f}$ face towards, it must be within at least one or two sectors simultaneously. Due to the sufficient condition, there is at least one node *S* falling in each sector and covering point *P* and $angle\left(\vec{f},\vec{PS}\right)\leq \theta$ holds. *T_k_*_+1_ performs all the same as other sectors here. If properly deployed, $\left\lceil \frac{2\pi }{\theta }\right\rceil$ nodes are enough to achieve full view coverage of a point.

**Figure 3:**
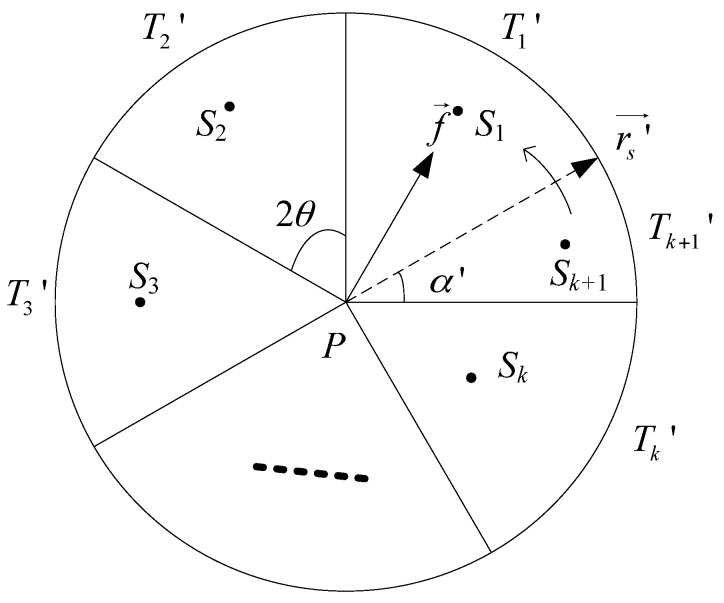
The sufficient and necessary condition of full-view point coverage with a minimum number of camera nodes.

It is redundant to deploy $\left\lceil \frac{2\pi }{\theta }\right\rceil$ nodes in *D*(*P*, *R*) for achieving full-view coverage of pint *P*. We turn to the sufficient and necessary condition of full-view point coverage with a minimum number of camera nodes. Rotate the start line $\vec{{r_{s}}^{\prime }}$ anti-clockwise with 2θ to get sector ${T_{1}}^{\prime },{T_{2}}^{\prime },...,{T_{k}}^{\prime }$ (shown in Figure 3), $k=\left\lfloor \frac{\pi }{\theta }\right\rfloor$. If $k<\frac{\pi }{\theta }$, then between ${T_{1}}^{\prime }$ and ${T_{k}}^{\prime }$ there may be one more sector ${T_{k+1}}^{\prime }$ with the interior angle $\alpha^{\prime }\in \left(0,\;2\theta \right)$. In the following explanation of sufficient and necessary condition, we agree on $k<\frac{\pi }{\theta }$.

The sufficient and necessary condition of full-view point coverage with a minimum number of camera nodes can be described as that at least one camera node locates in the angle bisectors of sector *T_j_* for all *j* = 1, 2, ···, *k*+1, which can be proved by contradiction.

Given the nodes *S*_1_, *S*_2_, …, *S_k_*, *S_k_*_+1_ locate in the angle bisectors of sectors and cover *P*, we analyze the full-view coverage with the different facing direction in the neighboring sector ${T_{1}}^{\prime }$, ${T_{k}}^{\prime }$, ${T_{k+1}}^{\prime }$. If *S_k_*_+1_ does not locate in the angle bisectors of the sector ${T_{k+1}}^{\prime }$, $angle\left(\vec{f},\vec{PS_{k+1}}\right)>2\theta$ must hold. It contradicts the condition for view-coverage. Therefore, the node must locate in the angle bisectors of the sector. If properly deployed, $\left\lceil \frac{\pi }{\theta }\right\rceil$ must be the minimum number of sensors to achieve full view coverage of a point. 

## 4. Density and Location Estimation for Deterministic Deployment

### 4.1. Dimension Reduction and Analysis

Deterministic deployment is to place nodes at planned, predetermined locations. When using deterministic deployment, it is often desirable to find a placement pattern such that the nodeS′ locations can be easily found. Furthermore, it is also desirable that such an arrangement pattern can achieve the lowest node density (the number of nodes per unit area) for complete coverage. 

In the previous section, we have gotten the lower requirement of nodes for full-view point coverage. It is infeasible to verify the full-view coverage of every point in a continuous domain. Hence, we proposed a novel method to develop the relationship between them. If a point is full-view covered by a set of camera nodes, the curious thing is whether there exist some nearby consecutive points full-view covered around the point. 

First of all, considering the geometric property of the coverage model, the maximum overlapped coverage region in the sector approximates to the inscribed circle in the sector. The maximum overlapped coverage area is disk D(P,r) with *P* as the origin and *r* as the radius. 

**Figure 4:**
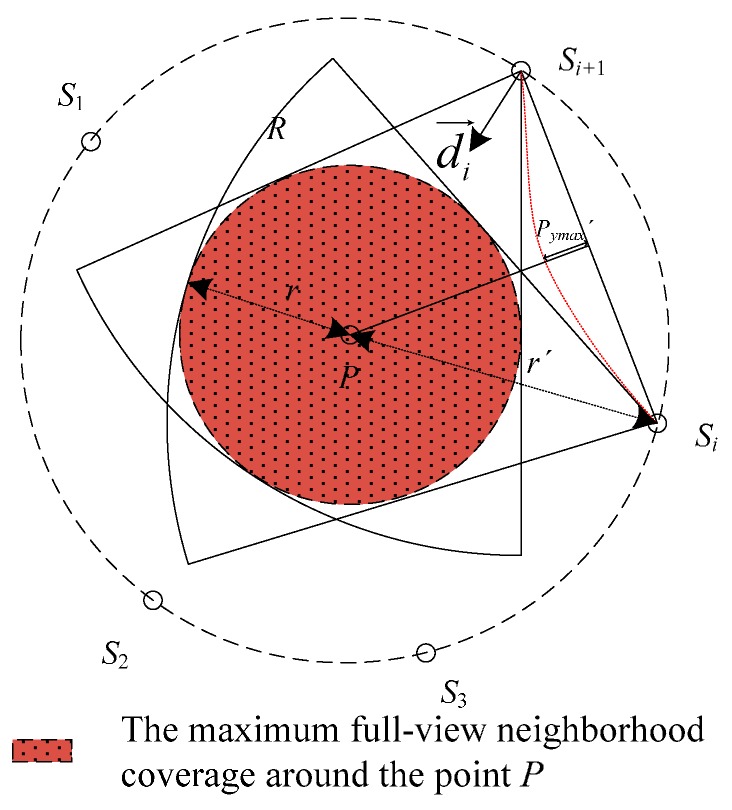
Full-view neighborhood coverage and the trajectory of nodes.

Besides, the trajectory of node positions can also be described as a circumference $C\left(P,r^{\prime }\right)$ with *P* as the origin and *r*′ as the radius, where $r=\frac{\sin\varphi }{1+\sin\varphi }R$, $r^{\prime }=\frac{1}{1+\sin\varphi }R$. The full-view neighbor-hood coverage and the trajectory of nodes shown in Figure 4 are presented as *D*(*P*, *r*) and *C*(*P*, *r′*) under a specified condition.

Combined with the necessary and sufficient conditions of full-view point coverage with the minimum number of nodes, we can estimate the optimal nodeS′ positions under deterministic deployment. The working directions of the nodes face towards the point by default. The first working direction starts from −180° and the next working direction rotates clockwise 2θ, and so on. If the location *P* (*P_x_*, *P_y_*) has been known, the position of *S_k_* can be described as in Equation (1):

(1) $$\left\{ \begin{array}{l} S_{kx}=P_{x}+r^{\prime }\cos(k\cdot 2\theta )\\ S_{ky}=P_{y}+r^{\prime }\sin(k\cdot 2\theta ) \end{array}\right.,\;k=1,2,...,\left\lceil \frac{\pi }{\theta }\right\rceil$$

**Figure 5:**
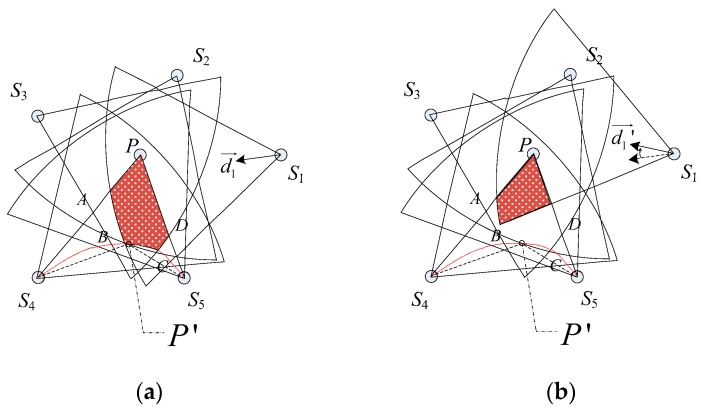
The example for interpreting the influence of the critical trajectory *P*′.

However, whether the disk D(P,r) is full-view covered is also subject to the distance between the adjacent nodes. To facilitate the analysis and design, we illustrate the constraint condition for full-view neighborhood coverage with the following instances. As showed in Figure 5a, *P* has been full view covered by *S*_1_, *S*_2_, *S*_3_, *S*_4_, *S*_5_. According to the geometric symmetry, we focus on the overlapped coverage region in $\Delta S_{4}PS_{5}$.

**Theorem.**  *With the same group of nodes, the maximum full-view neighborhood coverage around the point can be guaranteed if and only if* $\theta \in \left(0,\frac{\pi }{4}\right)$.

**Proof:**  If *P*′ can also be full-view covered by the same set of nodes, *P*′ must locate in the overlapped coverage region and $angle\left(\vec{P^{\prime }S_{4}},\vec{P^{\prime }S_{5}}\right)$ is less than or equal to the effective angle. We establish the Cartesian coordinates system with *S*_4_*S*_5_ as the horizontal axis and the perpendicular bisector of *S*_4_*S*_5_ as the vertical axis to draw the critical trajectory of *P*′, where the critical condition is $angle\left(\vec{P^{\prime }S_{4}},\vec{P^{\prime }S_{5}}\right)=2\theta$. The relationship of ${P^{\prime }}_{x}$ and ${P^{\prime }}_{y}$ is shown in Equation (2) as follows:

(2) $$\arctan\left(\frac{\frac{\left|S_{4}S_{5}\right|}{2}+{P^{\prime }}_{x}}{{P^{\prime }}_{y}}\right)+\arctan\left(\frac{\frac{\left|S_{4}S_{5}\right|}{2}-{P^{\prime }}_{x}}{{P^{\prime }}_{y}}\right)=\pi -2\theta$$

Taking the derivative of Equation (2), we have the extremum ${P^{\prime }}_{\max}(0,{P^{\prime }}_{y\max})$, where ${P^{\prime }}_{y\max}=\frac{\left|S_{4}S_{5}\right|}{2}\tan\theta$. The fact that *P*′ may intersect with the overlapped coverage region is determined by the working directions and the distance between the adjacent nodes. As shown in Figure 5b, as we rotate the working direction of *S*_1_ slightly, the critical trajectory of *P*′ has no influence on the full-view area coverage. The working directions all orientate towards the point *P* by default. Then we have the condition that $r+{P^{\prime }}_{y\max}<r^{\prime }\cos\theta$ for maximizing the full-view neighborhood coverage around *P*. We can simplify Equation (2) to Equation (3):

(3) $$\left|S_{i}S_{i+1}\right|<\frac{2R}{1+\sin\varphi }\left(\cos\theta -\sin\varphi \right)\cot\theta$$

(4) $$\left|S_{i}S_{i+1}\right|=2\left|PS_{i}\right|\sin\theta =\frac{2R\sin\theta }{1+\sin\varphi }$$

Substituting Equation (4) into Equation (3), we have Equation (5):

(5) $$\sin\varphi <\frac{2\cos^{2}\theta -1}{\cos\theta }$$

For $\varphi \in \left(0,\frac{\pi }{2}\right)$, Equation (5) can be rewritten as (6):

(6) $$\frac{2\cos^{2}\theta -1}{\cos\theta }>0$$

Therefore, we have the constraint condition $\theta \in \left(0,\frac{\pi }{4}\right)$ for achieving maximum full-view neighborhood coverage around *P*. □

At this point, the relationship between a point and its neighborhood has been established. Based on this, the full-view area coverage problem has been converted into the point coverage problem. Here the point is the center of the full-view covered disk. In the traditional disk model, triangle lattice-based deployment is proved to be optimal regarding sensor density [43]. In the next part, we establish the triangle lattice-based virtual grid points set for solving the full-view point coverage.

### 4.2. Deployment Pattern Algorithm for Deterministic Implementation

**Figure 6:**
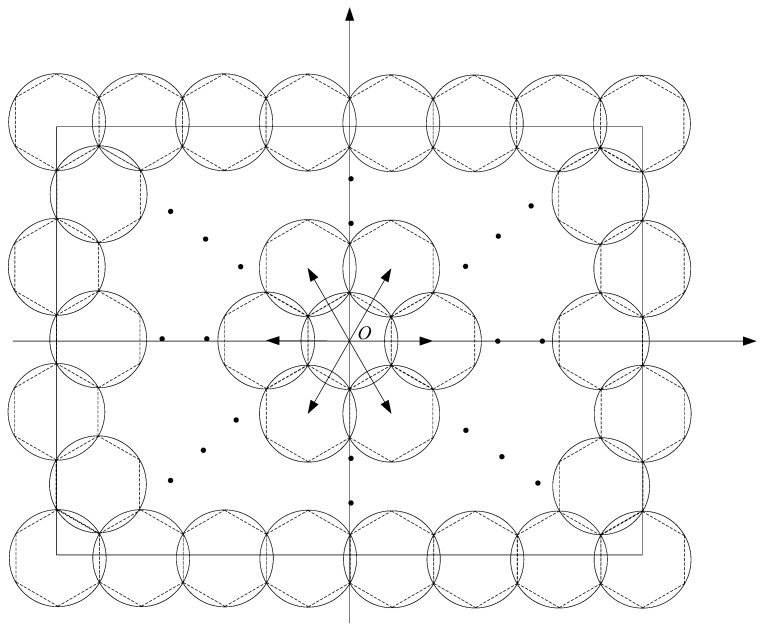
The diffusion method for ascertaining the positions of camera nodes.

In this section, we construct a deployment pattern algorithm for full-view area coverage based on the triangle lattice, which is shown in Figure 6. The grid length *l* of the triangle is critical. Given the sensing radius *R* and the effective angle, we calculate the best *l* such that every point in ROI is full-view covered.

To avoid the influence of boundary effects, we choose the triangle lattice-based virtual grid with the method of spreading from the center of ROI (as shown in Figure 6), in which the locations of nodes around the center (*x*_0_, *y*_0_) of the ROI are calculated out first. The deployment activities may be executed by a team of robots [44,45].

The position of the triangle lattice-based virtual grid point for the other full-view disk can be represented as $\left(x,\;y\right)\in \left\{x,\;y|\left(x_{0}\pm \sqrt{3}la,y_{0}\pm 2lb\right),\left(x_{0}\pm \left(\frac{\sqrt{3}l}{2}+\sqrt{3}lc\right),y_{0}\pm \left(\frac{3l}{2}+2ld\right)\right)\right\}$, where $a\in N|a\leq \lceil \frac{\sqrt{\left\|A\right\|}}{2\sqrt{3}l}\rceil$, $b\in N|b\leq \lceil \frac{\sqrt{\left\|A\right\|}}{4l}\rceil$ $c\in N|c\leq \lceil \frac{\sqrt{\left\|A\right\|}-\sqrt{3}l}{2\sqrt{3}l}\rceil$, $d\in N|d\leq \lceil \frac{\sqrt{\left\|A\right\|}-3l}{4l}\rceil$.

**Figure 7:**
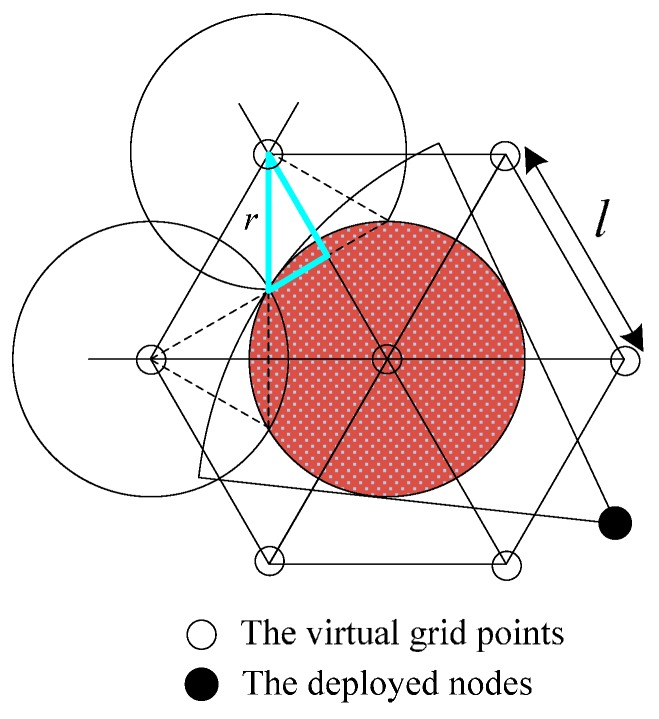
The triangle lattice-based seamless stitching method.

As shown in Figure 7, the optimal grid length can be computed based on the by the Pythagorean Theorem ($\cos\frac{\pi }{3}=\frac{r}{l}$). Thus, we get the optimal grid length l in Equation (7). For the truth that the minimum number of nodes for achieving full-view point coverage is $\left\lceil \frac{\pi }{\theta }\right\rceil$, the node density is $\left\lceil \frac{\pi }{\theta }\right\rceil$ times as likely to the density of the triangle lattice-based virtual grid points. The sensor density can be computed by the following Equation (8). Here, $\frac{3\sqrt{3}r^{2}}{2}$ is the area of the regular hexagon.

(7) $$l=\sqrt{3}r=\frac{\sqrt{3}R\sin\varphi }{1+\sin\varphi }$$

(8) $$\lambda =\frac{\#\left(the\;virtual\;grid\;points\right)}{A}\times \lceil \frac{\pi }{\theta }\rceil =\frac{2{\left(1+\sin\varphi \right)}^{2}}{3\sqrt{3}R^{2}\sin^{2}\varphi }\times \lceil \frac{\pi }{\theta }\rceil .$$

Deployment Pattern Algorithm: Given the region *A*, and the parameters of camera nodes. First, we establish the virtual grid points set (*x*, *y*) with the method of spreading from the center of the ROI. Specifically, the virtual grid points set can be obtained by the optimal grid length *l*. Then we solve the FACM problem by deploying the nodes around the virtual grid points gradually. For example, the nodes are deployed around the virtual grid point *P* with $r^{\prime }$. every 2θ to make sure the maximum full-view coverage in Figure 4. The process can be described by the pseudocode given as Algorithm 1.

**Algorithm 1. The Deployment Pattern Algorithm (DPA)**INPUT: the ROI *A*; the parameters of camera nodes (R, φ) OUTPUT: the optimal positions of camera nodes *S*1: Establish the virtual grid points set (*x*, *y*) from the center (*x*_0_, *y*_0_) of ROI
$$\;\left(x,\;y\right)\in \left\{x,\;y|\left(x_{0}\pm \sqrt{3}la,y_{0}\pm 2lb\right),\left(x_{0}\pm \left(\frac{\sqrt{3}l}{2}+\sqrt{3}lc\right),y_{0}\pm \left(\frac{3l}{2}+2ld\right)\right)\right\}$$ 2: For each point *P* in (*x*, *y*) 3: Deploy $n=\lceil \frac{\pi }{\theta }\rceil$ nodes around *P* with $r^{\prime }$ every 2θ
4: End

## 5. LNSA for Full-View Area Coverage in Random Deployment

The previous works concentrate on the derivation of the lower bound of the critical density that the ROI is full-view covered with random deployment. The critical density has been derived from the premise that the number of nodes tends to infinity. Here we take the same assumption that the redundant nodes are distributed in the ROI. Our goal is to find a minimum set of camera nodes guaranteeing the ROI can be full-view covered. According to the result derived from Section 4, a node scheduling algorithm is proposed to set the camera nodes into sleeping mode or working mode. For the reason that the scheduling process in the next round-robin schedule may be analogous to the first round, we primarily put the attention upon the first round of the scheduling process in our paper. 

**Figure 8:**
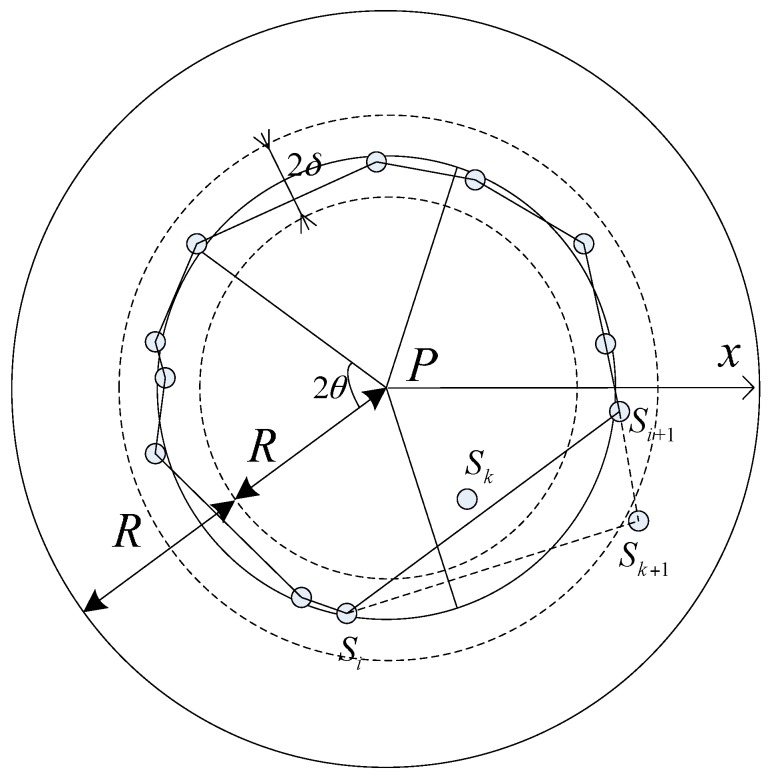
The local neighboring nodes in the disk $D_{i}\left(P_{i},2R\right)$.

As shown in Figure 8, the nodes are all set into the slightly sleeping model that the nodes can only send GPS signals. The algorithm starts when the node positions message has been confirmed and transformed to the remote console. The nodes randomly deployed in the boundary of the region may be sparser than in the interior area. The region is less likely to be covered as required due to the sparse density of nodes in the border. The conventional method of avoiding it is to deploy nodes in an expanded region. For ease of analysis, the activating progress proceeds with the diffusion process from the center of the ROI. According to Section 4, the ROI can be divided into many regular hexagons centered on the virtual grid where the scheduling algorithm is operated respectively.

As we mentioned above, the maximum full-view neighborhood coverage around the point *P* can be approximated to a disk D(P,r), and the position trajectory of nodes is a circle $C\left(P,r^{\prime }\right)$. For each regular hexagon, the FACM problem in each disk can be solved by selecting the camera nodes around the circle $C\left(P,r^{\prime }\right)$.

After dividing the ROI, the regular hexagon *H_i_* should be full-view covered by a minimum number of camera nodes. We schedule the nodes in the disk $D_{i}\left(P_{i},2R\right)$ with *P_i_* as center and 2*R* as the radius. The selected nodes will be deleted from the nodeS′ list. The polar coordinate system with *P* as the center and the radial *P_x_* as the polar axis is introduced because the key parameters discussed here are the angles between the adjacent nodes and the distances from *P_i_* to the nodes. In this section, the position of node *S_i_* can be represented as $\left(\rho_{S_{i}},\alpha_{S_{i}}\right)$, $\alpha_{S_{i}}\in \left(0,2\pi \right)$. 

It is assumed that *n* nodes *S* = {*S*_1_, *S*_2_, …, *S_n_*} exist on the disk $D_{i}\left(P_{i},2R\right)$. A threshold parameter δ∈(0,R) has been introduced to filter out the nodes far away from the critical trajectory. The nodes with $\left|\rho_{S_{i}}-R\right|\leq \delta$ are refined at first. Reordering the refined nodes with the increase of $\alpha_{S_{i}}$, the new node set can be represented as *S′_c_* = {*S_c_*_1_, *S_c_*_2_, …, *S _ck_*}. If the angle between the adjacent nodes, such as *S_cj_* and *S_cj_*_+1_, is greater than 2θ, the regular hexagon *H_i_* maybe not full-view covered. Therefore, the algorithm picks the spare nodes from $S-{S_{c}}^{\prime }$ to join *S′_c_* based on the nearest polar radius principle that the node whose polar radius is closer to *R* has more opportunity to be selected. 

The node set *S′_c_* around the critical trajectory has been achieved when there are no adjacent nodes that $\alpha_{{S^{\prime }}_{\mathrm{mod}(i+1,c)}}-\alpha_{{S^{\prime }}_{i}}>2\theta$. Next, the algorithm chooses nodes at intervals of 2θ as the awakening nodes in *S′_c_*. The specific process of the algorithm can be described by the pseudoicode of Algorithm 2.

**Algorithm 2.** The Local Neighbor-optimal Selecting Algorithm (LNSA)INPUT: the position information of all nodes *S* = {*S*_1_, *S*_2_, …, *S_n_*} in $D_{i}\left(P_{i},2R\right)$. OUTPUT: the selected nodes *S_b_* = {*S′_b_*_1_, *S′_b_*_2_, …, *S′_bi_*, …, *S′_bh_*}. 1: Divide ROI into many regular hexagons *H*_1_, *H*_2_, …, *H_i_*, …, *H_a_* with the virtual grid points as the center, build the polar coordinate system in *H_i_* 2: Perform the following steps in each regular hexagon *H_i_* 3: Refine the nodes in *H_i_* with $\left|\rho_{S_{i}}-R\right|\leq \delta$ and constitute *S′_c_* = {*S_c_*_1_, *S_c_*_2_, …, *S_ck_*} 4: for *i* = 1: *k* 5: If $\alpha_{{S^{\prime }}_{\mathrm{mod}(i+1,c)}}-\alpha_{{S^{\prime }}_{i}}>2\theta$ 6: Pick the spare nodes from $S-S_{C}^{\prime }$ to join *S′_c_* 7: *i* = *i* − 1 8: End 9: End 10: Choose nodes every 2θ as the awakening nodes *S′_bi_* = {*S_b_*_1_, *S_b_*_2_, …, *S_bm_*} in *S′_c_*.11: End 12: *S_b_* = {*S′_b_*_1_, *S′_b_*_2_, …, *S′_bi_*, …, *S′_bh_*}

## 6. Performance Evaluation

In this section, we perform simulations to verify that how many nodes are needed to achieve full-view coverage for the ROI. We consider two conditions as follows: in one case, the problem is how to design a deployment pattern algorithm such as that the ROI can be full-view covered by the minimum number of camera nodes. And in the other case, there are redundant camera nodes distributed in the ROI. The problem is how to select the minimum number of nodes for achieving the full-view area coverage. To our best knowledge, there are few studies on full-view area coverage with a minimum number of nodes, especially in computing the exact positions of camera nodes under the deterministic implementation. Therefore, we mainly compare our deployment pattern algorithm (DPA) and node scheduling algorithm (LNSA) with [19,36]. 

As the authors did not name their algorithms in their papers, we adopt Acronym Creator (http://acronymcreator.net/) to name the selected algorithms for comparison. Therefore, the algorithm presented by Wang [36] can be named as FURCA (for FUll-view Region Coverage Algorithm). The algorithm presented by He [19] can be named as DASH (for Dimension reduction And near-optimal Solutions).

### 6.1. Simulation Configuration

The ROI is a 100 m × 100 m square field. We use two settings for the sensing radius: *R* = 5 m and *R* = 15 m. In both situations, we deploy the nodes in the extended ROI with the ROI of (100 + 2*R*) m × (100 + 2*R*) m to avoid the boundary effect. The angle of view is fixed to be $\varphi \in \left(\frac{\pi }{6},\frac{\pi }{3}\right)$, and the effective angle is also fixed to be $\theta \in \left(\frac{\pi }{6},\frac{\pi }{4}\right),$ respectively.

We first show the relationship between the minimum number of nodes needed for full-view area coverage and the effective angle in the deterministic implementation with different sensing radius and angle of view.

In the second step of the simulation, we vary the number of nodes from 500 to 4500 for *R* = 15 with the different angle of view to observe the relationship between the critical density and the percentage of full-view area coverage. Besides, the number of selected nodes for achieving full-view area coverage is shown in the next section. we run ten times and take the mean to eradicate any discrepancies in the random deployment.

### 6.2. Simulations Analysis of DPA 

**Figure 9:**
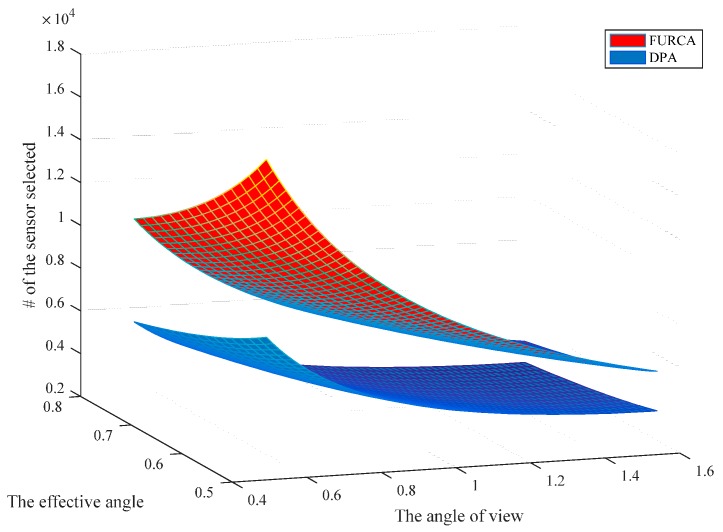
The direct-vision comparison of two deployment patterns.

We investigate the impact of the parameters on the performance of two deterministic deployment patterns by varying the value of the angle of view and the effective angle, respectively. The simulation results with *R* = 5 are shown in Figure 9. It provides an overview about the minimum number of sensors for achieving full-view area coverage. We notice that the number of sensors needed in our pattern is strictly smaller than the deployment pattern of FURCA. DPA select the less number of nodes than FURCA for the reason that we deduce the best gird length l with more specific condition compared with FURCA. FURCA obtained the best gird length l by contradiction, which does not consider the full-view coverage. DPA establish the internal relation between the full-view point coverage and the full-view neighborhood coverage.

**Figure 10:**
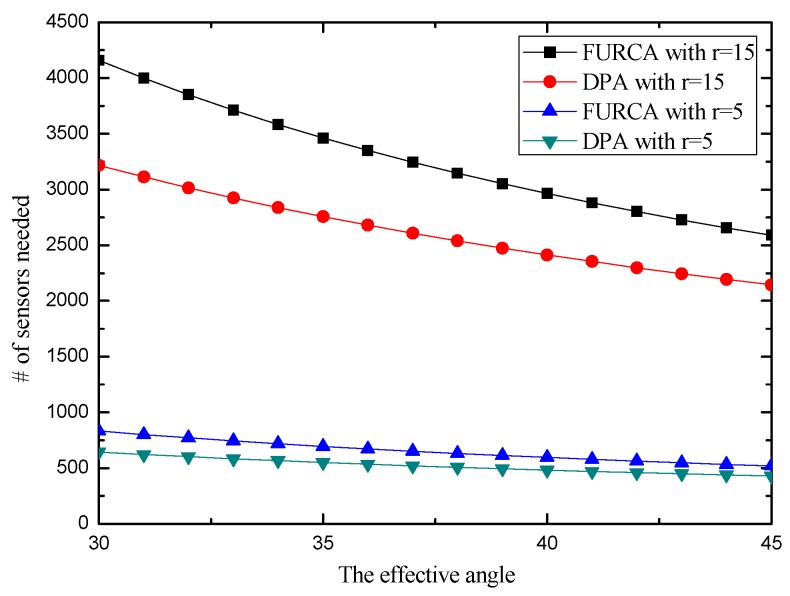
The number of sensors required vs. the effective angle.

**Figure 11:**
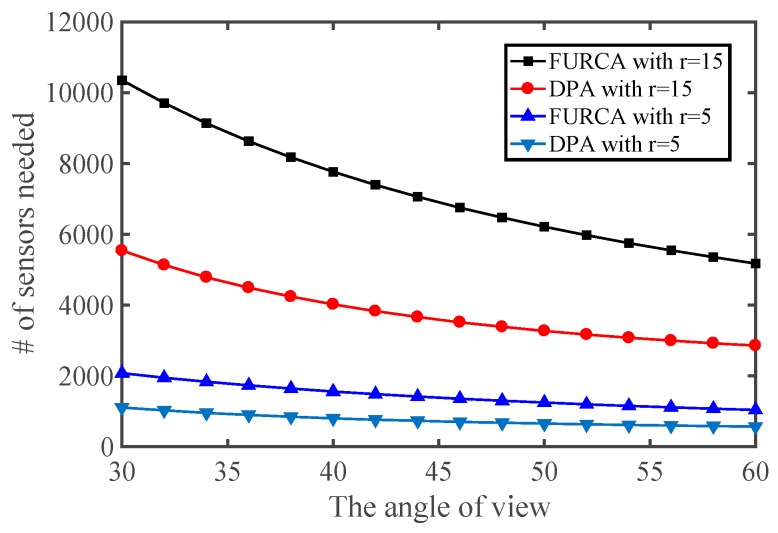
The number of sensors required vs. the angle of view.

In addition, the number of sensors required non-linearly decreases with the effective angle and the angle of view both in Figure 10 and Figure 11. This is because more targeted actions have been taken in the triangle lattices-based seamless stitching method in the proposed approach. However, the number of sensors required may be similar in both two kinds of deployment patterns when the sensing radius is small enough, which indicates that the proposed approach is restricted by the sensing radius. 

### 6.3. Performance Evaluation of LNSA

We design the probability of full-view area coverage for the ROI to describe the convergence performance of the scheduling algorithm. We assume that there are adequate virtual targets *t_a_* (# of all the targets) located in the ROI, where *t_s_* (# of the targets meeting conditions) can be full-view covered by the deployed sensors. The probability of full-view area coverage can be computed as $p=\frac{t_{s}}{t_{a}}$. It is evident that *p* may be close to the real probability of full-view area coverage for the ROI when *t_a_* tends to infinity. The Intruder in Sector (IIS) test works for computing the full-view coverage ratio. We perform IIS test in each virtual grid point and build the binary coverage array $A_{t_{a}}$.

(9) $$A_{i}=\{\begin{array}{c} 1,\;\mathrm{if}\;P\in Sc\;\mathrm{and}\;angle\left(\vec{f},\vec{PS}\right)\\ 0,\;\mathrm{else}\; \end{array},\;i=1,\;2,\;\ldots ,{\;t}_{a},\;\vec{f}\in \left(0,\;2\pi \right)$$

(10) $$t_{s}=\overset{t_{a}}{\underset{i=1}{\sum }}A_{i}$$

**Figure 12:**
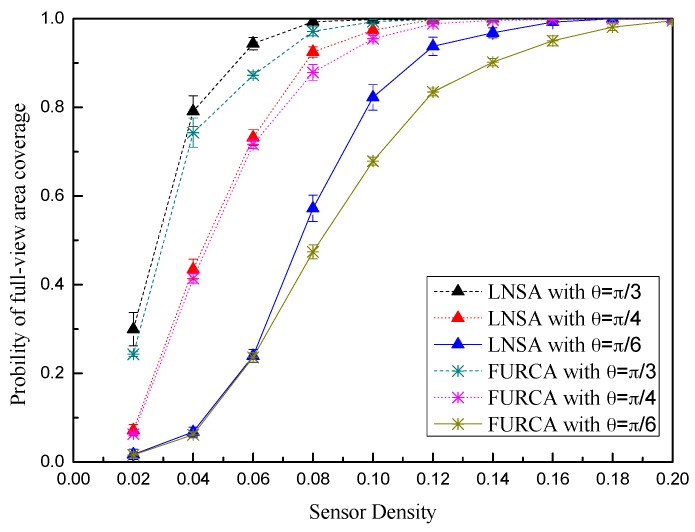
The probability of full-view area coverage vs. the sensor density.

We now investigate the convergence performance of the node scheduling algorithms, namely, FURCA and LNSA. The results are shown with the form of the error bars in Figure 12. We can see that the convergence rate of LNSA is faster than the convergence rate of FURCA during the middle and later periods. It is because that our scheduling algorithm can also adjust the working direction towards the triangle lattice points. With the growth of θ, the probability of full-view area coverage for the same node density also increases both in two scheduling algorithms. 

**Figure 13:**
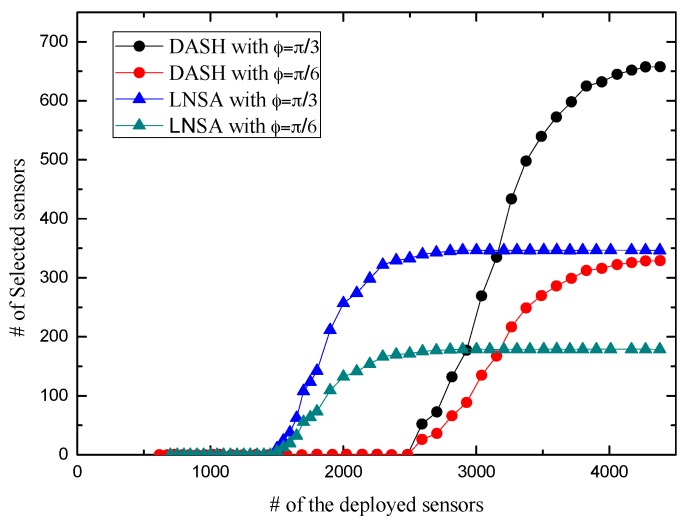
The number of selected sensors vs. the number of deployment sensors.

Then we proceed to investigate the relationship between the number of selected nodes and the number of deployed sensors. Here the sensing radius is fixed to *R* = 15. The results are shown in Figure 13. The beginning part in Figure 13 always tends to zero for the reason that the scheduling algorithms start work when the density of nodes increases to the quantity of insuring the whole region being full-view covered. In addition, our scheduling algorithm always goes ahead in the starting of the activating process. Besides, we find that the number of selected sensors of our scheduling algorithm is less than the one in DASH when the activating process finishes. It is because that our approach imposes restrictions on the working direction.

In the random deployment, FURCA proposed the probability that a given region A is full-view covered which indicates the full-view coverage status of ROI. It just guarantees ROI be full-view covered by increasing the number of nodes rather than adjust the working directions or scheduling the sleeping nodes. Based on the work of FURCA, DASH reduce the dimension of full-view area coverage by full-view ensuring set (FVES). DASH guarantees ROI be full-view covered while scheduling the sleeping nodes to prolong the lifetime of the networks. For the reason that LNSA adjust the working directions and scheduling the sleeping nodes, we have better simulation results in comparison with FURCA and DASH.

## 7. Conclusions

In this paper, we study the coverage issue of camera sensor networks. Different for the traditional binary sector coverage model, we are interested in the face direction of the target in the ROI. A model called full-view area coverage was introduced in this paper. With this model, we proposed an efficient deployment pattern algorithm DPA on the deterministic implementation for achieving the full-view area coverage with a minimum number of camera nodes. We also derived a node scheduling algorithm LNSA aiming to reduce the redundancy in a random uniform implementation. We investigated the performance of our deployment pattern with FURCA in term of the effective angle. The scheduling algorithms of DASH and LNSA were compared in term of the convergence performance and the number of selected sensors. Extensive simulation results validate the feasibility of the proposed algorithms. Although the coverage issue of full-view coverage is an emerging research field, further study may focus on the improvement of the sensing model and the verification of the algorithms when applied to specific applications. 

## Reference

## Figures and Tables

**Table 1:** Comparison between our work and the past related works.

Reference	Algorithm	Primary Objective	Main Contribution
[36]	FURCA	Full-view area coverage	The safe region and unsafe region
[18]	-	Finding critical condition of full-view area coverage	Equivalent sensing radius (ESR)
[38]	-	Full-view area coverage	Model the realistic sea surface
[19]	DASH	Full-view area coverage	Dimension Reduction
[37]	-	Finding critical condition of full-view area coverage	Critical sensing area (CSA)
This work	DPA/LNSA	Full-view area coverage	Maximum full-view Neighbor-hood coverage

**Table 2:** Notation used in this paper.

Symbol	Meaning
*S*	Camera node set, *S* = {*S*_1_, *S*_2_, …, *S_n_*}, where *S_i_* also represent the position of the *i*-th camera node
*P*	Location of the intruder
*R*	Sensing radius of the camera node
*r*	Radius of the maximum full-view neighborhood coverage disk
*r′*	Radius of the trajectory for nodes around *P*
φ	One-half of camera’s angle of view
θ	Effective angle
*l*	Grid length in the triangle lattice-based deployment
*λ*	Sensor density for achieving full-view area coverage
*Sc*	Camera’s field of view (FoV)
*D*(*P, r*)	Disk with *r* as the radius and *P* as the center
*T_k_*	The *k*-th sector
$\vec{d_{i}}$	Working direction of the *i*-th camera node
$\vec{f}$	Facial direction of the intruder
$\vec{r_{s}}$	Start line for dividing *C*(*P*, *R*) into *T*_1_, *T*_2_, …, *T_k_*
